# Editorial for Special Issue: Fungal Biology and Interactions

**DOI:** 10.3390/microorganisms12020282

**Published:** 2024-01-29

**Authors:** Roberto N. Silva, Renato Graciano de Paula

**Affiliations:** 1 Molecular Biotechnology Laboratory, Department of Biochemistry and Immunology, Ribeirao Preto Medical School (FMRP), University of Sao Paulo, Ribeirao Preto 14049-900, São Paulo, Brazil; 2 Department of Physiological Sciences, Health Sciences Centre, Federal University of Espirito Santo, Vitoria 29047-105, Espírito Santo, Brazil; renato.paula@ufes.br

As we conclude this Special Issue on fungal biology and interactions, it is only appropriate to reflect on the remarkable progress our scientific community has made in unraveling the mysteries of the fungal kingdom. From the intricate mycelial networks beneath our feet to the complex symbiotic relationships that shape ecosystems, the various facets of fungal biology have fascinated researchers and enthusiasts alike.

In recent years, fungal research has experienced an unprecedented upsurge, revealing the astonishing variety of functions that fungi play in different ecosystems. This Special Issue aims to consolidate and illuminate these findings and provides a platform to fill gaps in our understanding of fungal biology. The authors have compiled a rich body of knowledge that sheds light on the diverse interactions between fungi and their environment, including plants, animals, and other microorganisms.

One of the main objectives of this Special Issue was to fill critical knowledge gaps that still exist in the field of fungal biology. The articles presented in these pages provide invaluable insights into the molecular mechanisms that control fungal pathogenicity, the role of fungi in nutrient cycling, and the intricate web of interactions between microbes and the environment. By exploring these intricacies, researchers have made an important contribution to our understanding of the fundamental processes that govern fungal life.

The articles in this Special Issue address different facets of fungal biology, from transcriptome and metabolome analyses in response to infection, as shown by Li et al. (contribution 1), to the exploration of novel functions of cerato-platanin EPL2 in *Trichoderma reesei* (contribution 2), and the identification of chemical compounds in endophytic fungi from mangrove ecosystems (contribution 3). These diverse studies have contributed significantly to our understanding of fungal physiology, its interactions, and the potential applications of fungi in biotechnological fields, particularly in the production of enzymes (contribution 4).

However, even as we celebrate these achievements, it is important to recognize the gaps that still exist in our knowledge. The elusive nature of certain fungal species, the complexity of their interactions, and the dynamics of ecosystems pose challenges that need to be further explored. The search for a comprehensive understanding of fungal biology must continue, driven by curiosity and the realization that each new discovery opens new doors for research.

The future of fungal research offers exciting prospects. The integration of cutting-edge technologies such as genomics, transcriptomics, metagenomics, and advanced imaging techniques promises to reveal the hidden facets of fungal life [1]. The integration of omics technologies, as demonstrated by Yan et al. (contribution 5) in studying the effects of high temperatures on *Cenococcum geophilum* and by Jia et al. (contribution 6), provides a blueprint for future studies on fungal adaptability to environmental stressors. Interdisciplinary collaboration is crucial in deciphering the complex interactions between fungi and their effects on ecological stability and human well-being. Exploring the potential applications of fungi in sustainable practices, such as the development of environmentally friendly adsorbents by Tagyan et al. (contribution 7), highlights the broader importance of fungal research in addressing environmental problems. In addition, the dynamic field of mycorrhizal research, as explored by Prado-Tarango et al. (contribution 8) in the context of drought and competition, opens avenues for understanding the intricate relationships between fungi and plants in changing ecosystems.

Future research should prioritize deciphering the genomic intricacies of previously understudied fungal species, investigating the potential applications of fungi in biotechnology and medicine, and exploring the effects of climate change on fungal communities [2]. In addition, a deeper understanding of the ecological role of fungi in shaping plant–microbe interactions and nutrient cycling is essential for sustainable agriculture and ecosystem management [3].

In summary, this Special Issue is the result of a collective effort to push the boundaries of our knowledge of fungal biology. It serves as a springboard for the scientific community to embark on a journey toward a more comprehensive understanding of these enigmatic organisms. As we look to the future, let us not cease unraveling the mysteries of the fungal kingdom, fostering collaboration, and embracing the challenges that lie ahead in this ever-evolving field.

## Data Availability

Not applicable.
